# MINE: Module Identification in Networks

**DOI:** 10.1186/1471-2105-12-192

**Published:** 2011-05-23

**Authors:** Kahn Rhrissorrakrai, Kristin C Gunsalus

**Affiliations:** 1 Center for Genomics and Systems Biology, Department of Biology, New York University, 1009 Silver Center, 100 Washington Square East, New York, NY 10003, USA

## Abstract

**Background:**

Graphical models of network associations are useful for both visualizing and integrating multiple types of association data. Identifying modules, or groups of functionally related gene products, is an important challenge in analyzing biological networks. However, existing tools to identify modules are insufficient when applied to dense networks of experimentally derived interaction data. To address this problem, we have developed an agglomerative clustering method that is able to identify highly modular sets of gene products within highly interconnected molecular interaction networks.

**Results:**

MINE outperforms MCODE, CFinder, NEMO, SPICi, and MCL in identifying non-exclusive, high modularity clusters when applied to the *C. elegans *protein-protein interaction network. The algorithm generally achieves superior geometric accuracy and modularity for annotated functional categories. In comparison with the most closely related algorithm, MCODE, the top clusters identified by MINE are consistently of higher density and MINE is less likely to designate overlapping modules as a single unit. MINE offers a high level of granularity with a small number of adjustable parameters, enabling users to fine-tune cluster results for input networks with differing topological properties.

**Conclusions:**

MINE was created in response to the challenge of discovering high quality modules of gene products within highly interconnected biological networks. The algorithm allows a high degree of flexibility and user-customisation of results with few adjustable parameters. MINE outperforms several popular clustering algorithms in identifying modules with high modularity and obtains good overall recall and precision of functional annotations in protein-protein interaction networks from both *S. cerevisiae *and *C. elegans*.

## Background

Many types of molecular and functional associations, such as protein-protein or genetic interactions, can be usefully combined and represented as networks using graphical models. Understanding how molecular complexes and groups of functionally related gene products, or "modules", are organized within molecular interaction networks - both physically and in terms of functional dependencies - can lead to a better understanding of how cellular and developmental processes are coordinated. Because gene products within complexes or modules are expected to physically interact more frequently and to show stronger functional dependencies with each other than with other molecules in their environment, they are expected to share many more linkages in any network representation of functional associations. Topological analysis of network graphs can identify densely interconnected regions, which often correspond to functionally related groups of genes or proteins that can be identified as molecular complexes and modules, and can also reveal how different modules may be functionally linked.

Several algorithmic approaches have been developed to identify densely interconnected groups of vertices (also called nodes; here, genes/proteins) within a graph (here, biological interaction network). These can be broadly classified as *agglomerative *methods that grow clusters nucleated from densely interconnected regions (e.g. MCODE [1], CFinder [2], NeMo [3], SPICi [4]), or *divisive *methods that partition graphs into regions of differing connectivity (e.g. MCL [5]). Some general features differ between these approaches: for example, divisive methods usually attempt to assign all nodes in a graph into some cluster, while agglomerative methods do not; some methods assign nodes exclusively to a single cluster, while others allow membership of a single node in multiple clusters. We describe these five methods briefly below. MCODE is a popular clustering method that uses vertex weighting (a form of the clustering coefficient [6]) to grow clusters from a starting vertex of high local weight by iteratively adding neighboring vertices with similar weights. Cluster boundaries can be adjusted using options to trim vertices linked by a single edge ('haircut') or to draw in additional neighboring vertices ('fluff'). These options can allow nodes to remain unassigned or to be included in multiple clusters -- both likely scenarios *in vivo*, where the precise composition of functional modules and pathways may vary in different biological contexts. CFinder is a clique-finding algorithm that identifies fully connected subgraphs of different minimum clique size, and then merges cliques based upon their percentage of shared members, so that each node typically assumes membership in an entire hierarchy of clusters of differing sizes. CFinder results vary widely with each increment of minimum clique size (an adjustable parameter). NeMo identifies frequent dense subgraphs in input networks based on SPLAT [7] and CODENSE [8], which look for recurrence of dense subgraphs and coherent edge recurrence across subgraphs, respectively. NeMo is designed for dense, large-scale networks because it uses coherent edge frequencies, which can lose statistical power in sparse networks with few edges. MCL is a Markov Clustering method that is based on a flow simulation (essentially a random walk) that partitions a graph into areas of high and low flow. Nodes are grouped together as complexes when edges that link them have similar 'flow', or probability of edge use based on path. SPICi is a computationally efficient, local network-clustering algorithm that emphasizes optimizing cluster density. SPICi seeds clusters with nodes according to their weighted degree and accounts for local density around the growing cluster with each iteration. SPICi is promoted for its speed and ability to process large networks.

We applied all of these methods to molecular interaction networks from *Sacchromyces cerevisiae *(yeast) and *Caenorhabditis elegans *(worm) and compared their performance with respect to the modularity, density, and size of clusters, as well as the total number of clusters identified and their ability to group genes with similar functional annotations. To be as fair as possible in all comparisons and tests, we used the final clustering output from each implementation exactly as it was provided to the user. For the yeast networks we achieved some success using all of these methods, but we found them not as well suited for the worm interactome: the clusters identified were highly variable in quality, and adjustable parameters could not accommodate the higher interconnectivity of the worm network to produce consistently sensible results. We found the yeast network to have slightly higher density overall than the worm network (2.58e^-3 ^for FYI vs. 9.19e^-4 ^for WI8), while its characteristic path length (the average shortest path between all pairs of nodes) was nearly double that of for worm (9.24 vs. 5.16). This indicates that nodes in the worm molecular interaction network are more highly interconnected, and consequently would be expected to manifest less modularity, or separation of distinct clusters from the rest of the network. As a result, the methods described above were unable to identify consistently high quality clusters. For example, different algorithms variously tended to recover low-density, stringy clusters (MCODE), produce many small subnetworks that were subsets of larger modules (CFinder), lacked suitable parameter adjustability (CFinder, NeMo), partitioned the network exhaustively leaving no unassigned nodes (MCL), or tended to generate numerous small, exclusive (non-overlapping) clusters (SPICi).

Here we describe Module Identification in Networks (MINE), an alternative method we have developed that can effectively identify functional modules in the *C. elegans *molecular interaction networks. MINE at once robustly identifies highly interconnected clusters that are biologically coherent, has the flexibility to handle many different types of networks, and contains a small number of adjustable parameters that can be optimized for different network topologies - all within a simple graphical user interface. MINE is an agglomerative clustering algorithm very similar to MCODE, but it uses a modified vertex weighting strategy and can factor in a measure of network modularity, both of which help to define module boundaries by avoiding the inclusion of spurious neighboring nodes within growing clusters. We have evaluated MINE as applied to interactomes from yeast and worm, and we show that it performs favorably with respect to modularity and density in comparison with other current methodologies.

## Results

### Overview of algorithm and design considerations

**Figure 1:**
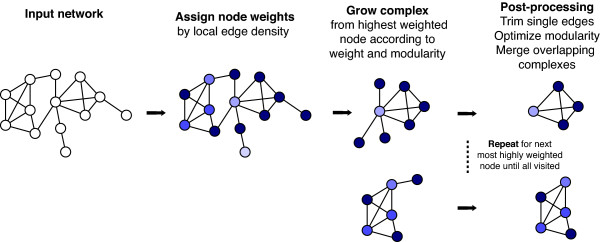
**Conceptual Overview of MINE Procedure**.

The clustering approach used by MINE is summarized in Figure 1 and Additional File 1 Figure S1. MINE first assigns weights to all nodes in a graph according to their edge degree and local neighborhood density. It then performs an iterative, agglomerative cluster finding procedure, in which clusters are seeded from nodes in order of their descending weight. With each iteration, the seed node is grouped together with neighboring nodes of similar weight and any neighbor nodes that improve the modularity score. After a cluster is delineated, it is compared to previously identified clusters and merged if there is significant overlap. This procedure is then repeated, starting with the next most highly weighted node, until all nodes have been inspected as a seed.

In developing MINE we reasoned that the algorithm should not attempt to force all vertices into a cluster, as it may not be feasible to assign every gene/protein to a physical complex or module in a real-world example - this may either reflect the underlying biological reality, or may occur because available network data is sparse and incomplete. We thus opted for an agglomerative clustering approach, and focused on three specific factors that are important for biologically and topologically meaningful cluster identification: neighborhood edge density calculation, optimization for modularity, and treatment of overlapping clusters. We discuss these three issues and their influence on performance separately.

#### Neighborhood edge density

To build clusters, MINE uses a strategy similar to that of MCODE, which we had found to return good results in yeast (but which did not provide the flexibility we sought for *C. elegans*). The primary differences lie in the method that MINE uses to calculate how vertices are weighted and the inclusion of a local modularity score at each step. To retain information about the precise local neighborhood of a vertex (all directly connected vertices, i.e. all connected vertices of depth 1), we assign the vertex (*v*) a weight (*v*_*w*_) that is the product of its own clustering coefficient, i.e. its density (*d*), and the number of edges (*k*) of the most highly connected node in the local neighborhood of *v*, inclusive of *v *(*k*_*max*_):

This weighting scheme improves the scores of densely grouped genes that are linked to a highly connected node, or 'hub'. The topological effect of this scoring scheme is to place higher weight on vertices connected to hubs, which have been shown to be important for robustness in biological interaction networks and tend to occur within functional modules [9].

#### Modularity

We include an additional parameter that takes into account a modularity score, which represents the level of connectivity within a group of nodes relative to the group's connections to the rest of the network. Modularity is defined as the ratio of the number of edges between nodes in a cluster (in-degree, *E*_*in*_) to the number of edges between members of the cluster and any neighbors not designated as members of the cluster (out-degree, *E*_*out*_):

A high modularity score will indicate that a cluster is very isolated from the rest of the network. Thus in expanding a cluster, not only is the weight of a vertex considered, but also whether its inclusion will improve the modularity score. Thus, nodes that satisfy the vertex weight threshold but which *decrease *the modularity score by more than *ΔC*_*mod *_are *not *added; conversely, nodes that *improve *the modularity score of the cluster by at least *ΔC*_*mod *_*are *added, even if they do not satisfy the vertex weight threshold. Finally, all clusters undergo an iterative culling procedure that removes nodes if this will increase the score of the remaining cluster by at least *ΔC*_*mod*_. *ΔC*_*mod *_is implemented as the user-specified parameter *msp *(modularity score percentage).

#### Overlapping clusters

One of the attractive features of CFinder is its ability to recover overlapping clusters, which is compatible with the idea that complexes in a biological system are not necessarily static; all or part of a complex may be activated at a specific time or location, and component parts may even be included in multiple complexes. Clusters identified algorithmically should reflect this property, and thus we designed MINE so that it can return both exclusive and non-exclusive clusters, and can merge together clusters that appear to overlap above a user-defined threshold (with the default set at 50% shared nodes). Among all the algorithms we compared, CFinder is the only other method that is able to cluster while permitting overlaps; however in contrast to CFinder, MINE has been designed to avoid returning both the parent and child clusters (clusters that are primarily a subset of a larger 'parent' cluster) where it would be more appropriate to combine them.

### Performance Evaluation

MINE was tested using protein-protein interaction data from *S. cerevisiae *and *C. elegans *and compared with the performance of five other algorithms. The yeast *S. cerevisiae *is a classic model organism for which a great deal is known about protein complexes, and thus presents an ideal opportunity to test a new network clustering algorithm. We used as our test networks all yeast two-hybrid data from BioGRID [10] and the 'Filtered Yeast Interactome' (FYI) [9], which represents very high confidence protein-protein interactions. For annotated complexes, we used MIPS [11] and GO-SLIM Macromolecular Complex annotations [12] as gold standards against which to measure complex identification within these networks. Clusters identified by MINE were then compared with annotated complexes contained in the yeast networks. For *C. elegans*, we used protein-protein interaction networks based on WI8 [13], as well as all physical interactions from both MINT [14] and IntAct [15]. In contrast to yeast, *C. elegans *is a biologically more complex organism for which, despite its well-studied genetic and developmental networks, there is no well-annotated database of protein complexes. We used *C. elegans *Gene Ontology (GO) annotations for Biological Process, Cellular Component, and Molecular Function to provide a comparable validation set. Only GO terms with at least 3 and at most 100 members were considered to avoid categories that are too general or too specific. MINE was tested over a broad range of parameters for vertex weight percentage *vwp *(0 - 100%) and modularity score percentage *msp *(0 - 100%). Four of the five tested algorithms (CFinder, MCL, SPICi and MCODE) also include adjustable parameters and were evaluated across a wide spectrum of their settings. The performance of all algorithms was then assessed in terms of recall and precision, modularity, and geometric accuracy of identified clusters with respect to annotated complexes.

#### Recall and Precision

For both measures, all annotated complexes (according to MIPS or GO terms) were matched to predicted clusters with the most significant overlap as measured by the hypergeometric test (p-value ≤ 0.05). Recall is defined as the number of true positives (TP) over the sum of all true positives and false negatives (FN): Recall = TP/(TP+FN). Precision was calculated for the same cluster, and is defined as the number of true positives divided by the sum of true positives and false positives (FP): Precision = TP/(TP+FP). In both measures, true positives are defined as gene products that are annotated as members of a protein complex by either GO or MIPS.

**Figure 2:**
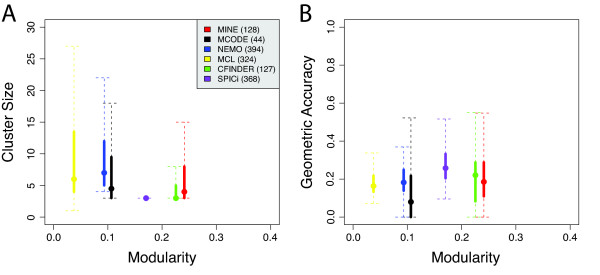
**Modularity vs. Cluster Size and Geometric Accuracy at Optimal Settings**. for each algorithm, we selected the setting with the optimal balance of modularity and average geometric accuracy for the *C. elegans *interactome from WI8 based on GO Cellular Component annotations. The boxplot, below, represents the global modularity of the clusters (x-axis) vs. A) the distribution of cluster sizes (y-axis) and B) the distribution of the geometric accuracy (y-axis). The circle indicates the median value; thick lines indicate upper and lower quartiles; whiskers indicate 1.5 times the inter-quartile range (IQR). The total number of clusters identified by each algorithm is indicated in parentheses in the key. A) The plot shows that MINE produces clusters of varying sizes while maintaining a higher overall modularity. B) The plot shows that MINE produces clusters with a much higher overall modularity and a similar range of geometric accuracy as other algorithms without producing an artificially large number of clusters.

In yeast, MINE was consistently among the top performing algorithms with respect to both recall and precision for capturing MIPS and GO complexes in both networks (Additional File 1 Figures S2A-D). When examining the higher density *C. elegans *interactome, MINE generally achieved a balance of recall and precision slightly higher than MCODE and CFinder when considering GO Molecular Function, Biological Process and Cellular Component (Additional File 1 Figures S2E-M). While MCL and SPICi can reach a higher precision and recall, they typically do so at the expense of producing many more (Additional File 1 Figures S3C-E) and/or generally smaller (Figure 2A and Additional File 1 Tables S1, S2) clusters than any of the other algorithms. Average precision and recall are inflated in these cases by the higher contribution of very small clusters, which necessarily have a lower bound on the proportion of potential false negatives and false positives when at least one node is a true positive (a requirement for inclusion in the composite score). Though there are parameter settings at which SPICi can perform better than other methods on the *C. elegans *protein interaction network, like most of the algorithms tested it does so with the constraint of identifying only exclusive clusters.

#### Modularity

We evaluated global cluster modularity using a measure defined in [16]. The global modularity score is calculated from a composite of the local modularity scores across all clusters and accounts for edges inside each cluster, edges connecting each cluster to the rest of the network, and the total number of edges in the network. The composite score provides a clear assessment of each algorithm's ability to delineate clusters that are well separated from the rest of the network.

When evaluated over a range of parameters, we find that MINE produces clusters with good separation from the rest of the network, and also produces more clusters of higher modularity than other methods, for both the yeast and worm interactomes (Additional File 1 Figure S3). In the yeast networks, MINE consistently outperforms other methods, with the exception of a single setting for CFinder and NeMo in the FYI network (Additional File 1 Figures S3A-B). For worm, only a single setting of CFinder achieve comparable modularity and total number of clusters identified by MINE (Additional File 1 Figures S3C-E); SPICi can produce higher overall composite modularity, but there is an insignificant difference between the distribution of modularity scores for SPICi and MINE (Figure 2A, Additional File 1 Table S2 and data not shown). Other algorithms also tend to produce a much greater variation in the total number of clusters identified across their parameter settings, while still producing clusters of lower modularity; this is particularly striking for MCL (Additional File 1 Figure S3).

#### Geometric Accuracy

**Figure 3:**
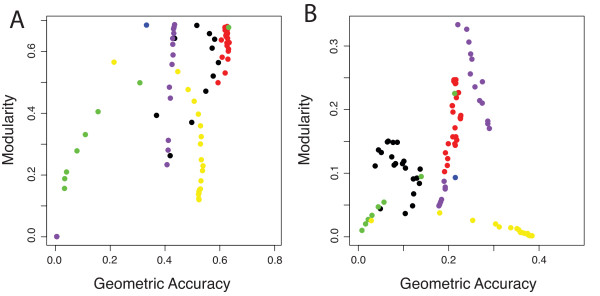
**Geometric Accuracy vs. Modularity of Predicted Complexes**. Plot of geometric accuracy against global modularity across a range of parameters for five algorithms: MINE (red), MCODE (black), NEMO (blue), CFinder (green), MCL (yellow), and SPICi (purple). See text for details on different algorithms. A) *S. cerevisiae *FYI network, evaluated using MIPS complexes. B). *C. elegans *interactome network from WI8, evaluated using GO Cellular Component annotations with 3-100 gene members.

Geometric accuracy simultaneously reports on the recall and precision of clustering performance, and is defined as the geometric mean of these two measures. This single score provides an effective measure for evaluating performance against annotation sets. Using the mean geometric accuracy of all clusters at different parameter settings, MINE consistently performs better than most other methods over a range of parameters, with a typical geometric accuracy of ~70% in yeast and ~22% in worms (Figure 3). Results from MCODE, MCL, SPICi and CFinder vary in geometric accuracy over a much wider range. When plotted against the composite modularity (Figure 3 and Additional File 1 Figure S4), MINE performs favorably with respect to topological separation from the network and the ability to identify high-quality clusters of varying sizes that capture commonly recognized biological modules.

## Discussion

**Figure 4:**
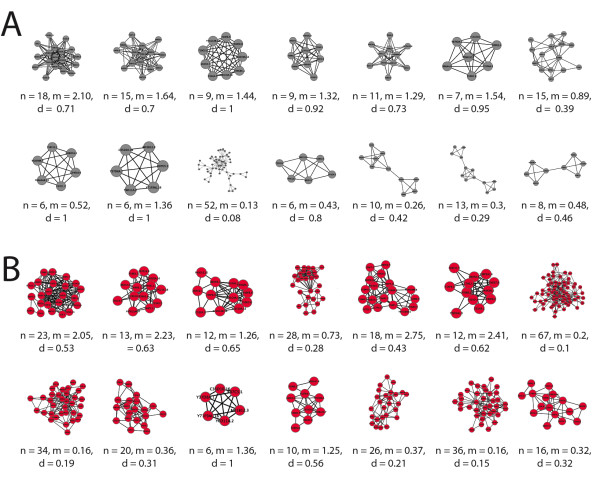
**Comparison of Top MINE and MODE Cluster Results**. Representative examples of cluster results from MCODE and MINE for the *C. elegans *interactome from WI8, showing the 14 highest-scoring clusters from each algorithm. For each method, parameters were chosen to provide the optimal balance between modularity and highest geometric accuracy for GO Cellular Component. Cluster size (*n*), local modularity (*m*), and density (*d*) are provided below each cluster. A) MCODE (*vwp *= 0.30; *haircut *= true). B) MINE (*vwp *= 0.90; *mod *= 0.30; *trim *= true).

For both yeast and worm interactomes, MINE surpasses other methods in recovering clusters that are well separated from the rest of the network, while achieving good recall of annotated complexes (Figure 3 and Additional File 1 Figure S4). Of the algorithms that do not allow cluster overlap, SPICi appears to have better performance with respect to mean geometric accuracy and composite modularity; it even is slightly higher than MINE with respect to these measures. However, MINE maintains comparable performance while allowing nodes to be shared between clusters, a feature that SPICi lacks. We consider this to be of high biological relevance in a multicellular organism like *C. elegans*, in which different functional modules are reused in different spatiotemporal contexts where their precise molecular composition may vary. Additionally, MINE results are robust to a variety of parameter settings and consistently identify high quality clusters with respect to the defined measures. This is in contrast to other methods, for which the user must test over a broad range of parameters to find the optimal setting. Thus, MINE offers a simpler tool for the end user to identify high quality clusters without the need for extensive optimization irrespective of any *a priori *knowledge of the network. MINE does show excellent performance when all six algorithms are compared at settings that provide an optimal balance between modularity, geometric accuracy, and cluster number in *C. elegans *WI8 (for GO Cellular Component, Figure 2B and Additional File 1 Table S1; the same is true for other GO categories, data not shown). Here again MINE is one of the top performers; its slightly lower modularity with respect to SPICi is the result of its cluster overlap feature. Moreover, if methods are compared at settings optimized solely for geometric accuracy (again, for GO Cellular Component), MINE remains one of the top performers with respect to modularity, geometric accuracy, mean cluster density and mean cluster size (Additional File 1 Table S2). This performance advantage is illustrated graphically in Figure 4, where the top fourteen clusters from MINE and MCODE (the most closely related algorithm to MINE) are displayed from an analysis of the *C. elegans *protein-protein interactome, using optimal parameters with respect to geometric accuracy and modularity for both algorithms. Clusters identified by MINE are more highly interconnected and less prone to comprise multiple distinct clusters of nodes that have been gathered together and reported as a single module; MCODE clusters progressively lose cohesiveness as cluster scores decrease.

We also note that MINE specifically filters for clusters that are of size 1 or 2, as those are too small to be considered valid groups of genes (in contrast to some other methods). This size criterion also accounts for some of the differences in coverage (i.e. total number of nodes clustered) between MINE and other methods. By eliminating clusters of size 1 and 2, many genes remain isolated, consistent with the biological intuition that not every gene can be clearly associated with a functional module in any particular dataset.

MINE performs very competitively with existing methods and offers a small number of tuneable parameters, rendering this method highly adaptable for different input networks. With an emphasis on graph-based clustering and modularity, MINE behaves well on both spare, modular networks and large, dense networks. In contrast to MCL, CFinder, SPICi and MCODE, the results produced by MINE do not change dramatically with small parameter adjustments, thereby offering the user both the ability to quickly discover high quality clusters and fine-grained control over the final set of clusters. This is likely because the evaluation of modularity for each vertex addition acts as a buffer that prevents large changes in cluster results. We found that MINE also outperformed most other methods when additional noise was introduced to test networks (data not shown). Across all methods, the geometric accuracy obtained for the worm interactome was significantly lower than for the yeast network. This is likely because the *C. elegans *interactome, although densely interconnected, still has relatively low coverage and is missing many known interactions [13]. Combined with the low coverage of GO annotations for the worm genome, the likelihood of recovering all components annotated with a given GO category is reduced relative to the comparatively well-annotated yeast genome.

## Conclusions

MINE is a highly tuneable graph-clustering algorithm whose strengths for the identification of molecular complexes are more pronounced in dense, highly interconnected networks, such as the *C. elegans *protein-protein interaction network. MINE uses a small number of adjustable parameters that enable it to identify high quality clusters that share common functional annotations. MINE is implemented both as a Cytoscape plug-in and a Perl script. The Cytoscape plug-in provides a simple graphical user interface (GUI), whereas the Perl version allows automated batch processing and offers several extensions to the core MINE package, which include: edge weighting, requiring vertex weights above background distribution for inclusion in a cluster, identification of vertices that act as linkers between clusters (non-clustered nodes that connect two non-overlapping clusters), and the ability to utilize expression or localization data to generate sub-networks for condition-specific cluster identification. These additional features position MINE as a particularly versatile tool for identifying the composition of functional modules within molecular networks.

## Methods

### Scoring

MINE receives as input any number of interaction files. The network is treated as an undirected, unweighted graph. All vertices *V *in the graph *G *= (*V*, *E*) are then weighted based upon their local neighborhood *N*, defined as the set all vertices connected directly to *v *(at a depth of 1); we call the set *N *inclusive of *v *itself {*N*∪*v*}, which we denote simply as *N*∪*v*. The vertex weight (*v*_*w*_) is the product of the maximal number of edges connected to any single node in *N*∪*v *(*k*_*max*_) and the density of *N*∪*v *(*d*): *v*_*w *_*= k*_max _* d. Density is calculated as *d = *2 *e*_*N*∪*v*_/(*V*_*N*∪v _* (*V*_*N*∪*v *_- 1)), where *V*_*N*∪*v *_is the number of vertices in *N*∪*v *(i.e. *v *and its direct neighbors) and *e*_*N*∪*v *_is the number of edges in *N*∪*v*. A cluster (*C*) is then established by iterating through each vertex in order of highest to lowest weight and adding neighbors if either of two criteria are satisfied: A) the neighbor vertex weight is above a minimum threshold (as determined by the user-defined vertex weight percentage (*vwp*) of the seed vertex) and does not decrease the cluster modularity score (by an amount equal to or greater than the user-defined modularity score percentage (*msp*)); B) the modularity score for the cluster is improved by *msp*. Cluster modularity (*C*_*mod*_) is defined as the ratio of edges between nodes of a cluster (*E*_*in*_) and edges between cluster members and non-members (*E*_*out*_): *C*_*mod*_*= E*_*in*_*/E*_*out*_. The process is continued exhaustively until no further vertices can be added, and is then repeated over all vertices in order of descending *v*_*w*_. Clusters are next evaluated for improvements of modularity scores if members are removed. They may optionally be refined further by removing all vertices with *k *= 1 (if the flag *Trim *is set). By default, clusters are non-exclusive (i.e. members are allowed to participate in several clusters), and clusters that overlap by > 50% are merged. A cluster is scored (*C*_*s*_) as the product of its density (*d*) and the number of members in the cluster (*V*_*C*_): *C*_*s *_= d * V_*C*_.

### Algorithm

#### 1. Vertex Weighting

procedure Vertex-Weighting

input: graph: *G *= (*V,E*)

for all *v *in *G*

*N *= set of immediate neighbors of *v *(depth = 1)

*k*_*max *_= maximum number of edges from any one vertex in set *N*∪*v*

*d *= density of *N*∪*v*

*v*_*w *_= weight = *k*_max _* d

end for

end procedure

#### 2. Cluster Prediction

procedure Cluster-Prediction

input: graph: *G *= (*V,E*); vertex weight: *v*_*w*_; vertex weight percentage: *vwp*; modularity score percentage: *msp*; merge percentage: *mp*

for *v *∈ *V*_*w *_(from high → low weight)

  push (*tocheck*, *v *)

  while *tocheck *not empty

    *n *= pop(*tocheck*)

    push (*visited*, *n*)

    *N *= set of immediate neighbors of *n *(depth = 1)

    if ( *n *== *v *)

      *v*_*s *_= *v*

    else

      *v*_*s *_= source vertex in cluster that pushed vertex *n *onto *toCheck*

    if *v*_*w *_of *n *≥ (*v*_*w *_of *v*_*s*_)(1 - *vwp*) then

      if modularity-score(*C*∪*n*) > modularity-score(*C*) - modularity-score(*C*)*msp then

      add *n *to cluster *C*

      push(*tocheck*, {*N*\{*C*∪*visited*}})

    else if modularity-score(*C*∪*n*) > modularity-score(*C*) + modularity-score(*C*)*msp

      add *n *to cluster *C*

      push(*tocheck*, {*N*\{*C*∪*visited*}})

    end if

  end while

  if trim == true then call: Trim (*C*)

  for *v *∈ *V*_*C*_

    if modularity-score({*C *\*v*}) > modularity-score(*C*) + modularity-score(*C*)*msp then

      remove *v *from *C*

  end for

  if percent overlap C with existing cluster ≥ *mp*

    Merge(*C*) with existing cluster

*C*_*score *_= density(*C*) * sizeof(*C*)

end for

end procedure

procedure Trim

input: cluster: *C*

for all *v *in *C*

if *k *of *v *< 2 then remove *v *from *C*

end for

end procedure

procedure modularity-score

input: cluster: *C*

*in *= number of edges exclusively between members of *C*

*out *= number of edges exclusively between members and non-members of *C*

score = in/out

end procedure

### Recall and Precision

Recall and Precision were calculated for each cluster with respect to all annotated complexes in the validation set (MIPS or GO ontology), and the complex showing the most significant overlap with the cluster was selected as the representative annotation for performance evaluations among different algorithms. For each annotated complex, true positives (*TP*) are defined as members of the annotated complex that are found in the cluster; false positives (*FP*) are defined as cluster members that are not part of the annotated complex; false negatives (*FN*) are defined as annotated complex members that are not part of the cluster. Recall is calculated as *TP/(TP + FN)*. Precision is calculated as *TP/(TP + FP)*. To arrive at an aggregate statistic, the mean recall and precision across all annotated complexes were calculated using the highest scoring cluster for each annotated complex. Significance was calculated using a hypergeometric test (p-value ≤ 0.05).

### Modularity

Global modularity was calculated according to [16] and [17]. This measure provides a composite modularity score across all clusters and is defined as:

where, for each cluster *c *in the set of all clusters *C*, *E*_*cIn*_, *E*_*cOut *_and *E*_*total *_represent the number of edges within the cluster, the number of edges leading out of the cluster, and the total edges in the network, respectively. We note that while the global modularity score only considers clusters that are contained within the main graph component, in practice this does not significantly affect the results because few or no clusters in the networks we consider are isolated from the main component. Local modularity for each cluster is defined as: *C*_*mod *_= *E*_*cIn*_/*E*_*cOut*_. The MINE algorithm uses only local modularity in predicting individual clusters, while the global modularity score serves as an aggregate statistic on the cumulative output.

### Geometric Accuracy

Geometric accuracy is defined as √(R * *P*), where *R *is Recall and *P *is Precision. This measures how well an algorithm is able to strictly identify a training set of complexes from the validation set without drawing in too many extraneous nodes.

### Algorithm Comparison

MINE was tested over a range 30 settings of *vwp *(0.1 - 1) and *msp *(0.1 - 1) with *trim single *edges = True. The MCODE Cytoscape plug-in was run with *haircut *= True and *depth *= 2 over 21 settings of of *vwp *(from 0 to 1). NeMo was executed with its Cytoscape plug-in and offers no adjustable parameters. CFinder was downloaded from http://angel.elte.hu/cfinder/ and tested with 8 *k *clique sizes ranging from 3 to 10. MCL was executed as the R package *mclR *(distributed by http://micans.org/mcl/) with 20 granularity settings ranging from 1.2 to 5.0. SPICi was downloaded from http://compbio.cs.princeton.edu/spici/ as a C++ distribution and tested for 20 density settings from 0.1 to 1.0.

### Datasets

For the network analysis, we used the following protein-protein interaction maps: for yeast, the Filtered Yeast Interactome *FYI *[9] and BioGRID yeast two-hybrid data [10]; for *C. elegans*, three datasets were used: 1) physical interactions from MINT [14], 2) physical interactions from IntAct [15], 3) a combined network of WI8 (Worm Interactome version 8) [13], supplemented with interologs (inferred interactions between orthologous proteins as identified by InParanoid from *D. melanogaster, S. cerevisiae*, and *H. sapiens*) [18], and a domain-based interaction map of proteins involved in embryogenesis [19]. We also evaluated the performance of MINE using WI8 only and obtained essentially the same results (data not shown).

Several training sets were used for validation: yeast MIPS annotated complexes (http://mips.gsf.de/genre/proj/genre), GO Macromolecular Complexes for *S. cerevisiae *and GO categories [12] for *C. elegans*. 127 MIPS complexes and 175 GO Macromolecular Complexes are present in the FYI map. 98 MIPS complexes and 209 GO Macromolecular Complexes are present in the yeast two-hybrid from BioGRID map and these were used for all validation in yeast. For validation in *C. elegans*, GO annotations from all three ontologies, Biological Process, Cellular Component and Molecular Function, were used. We considered only GO terms with at least 3 and at most 100 annotated members.

### Implementation and availability

MINE is available as a Cytoscape plug-in (compatible with versions of Cytoscape 2.4 and up) from the Cytoscape website (http://www.cytoscape.org) and can be installed and updated through the built-in plugin manager; it has also been provided as Additional File 2 and should be placed in the plugin folder of one's local Cytoscape installation. Finally a Perl implementation, which offers several extensions to the core MINE algorithm, is available from the authors upon request.

## Authors' contributions

KCG and KR conceived of the project, KR implemented and tested the algorithm, KCG provided guidance for the project, and KR and KCG wrote the paper.

## Supplementary Material

Additional file 1 **Supplementary Figures 1-4 and Supplementary Table 1 in PDF format**. Click here for file

Additional file 2 **MINE Cytoscape plugin**. Click here for file
