# Erratum to: MINE: Module Identification in Networks

**DOI:** 10.1186/s12859-016-0929-5

**Published:** 2016-02-17

**Authors:** Kahn Rhrissorrakrai, Kristin C. Gunsalus

**Affiliations:** 1 IBM Computational Biology Center, 1101 Kitchawan Rd., Yorktown Heights, NY 10598 USA; 2 Center for Genomics and Systems Biology, Department of Biology, New York University, 12 Waverly Place, New York, NY 10003 USA

## Erratum

It was brought to our attention that there was a discrepancy between the description and implementation of the MINE algorithm in our article [1]. We regret any inconvenience that may have resulted from this inaccuracy.

The primary difference is that as implemented, only the immediate neighborhood of each node is searched to identify initial clusters, and the merging process then facilitates cluster growth and removal of redundant clusters. This implementation results in accelerated cluster prediction relative to a more exhaustive search, which would perform comparably to a depth-first search across a larger portion of the network for each node. The published results and performance of MINE are unaffected; the description of the method, algorithm pseudocode, Fig. 1, and Additional file 1: Figure S1 have been updated to accurately reflect the implemented version of MINE.

**Fig. 1:**
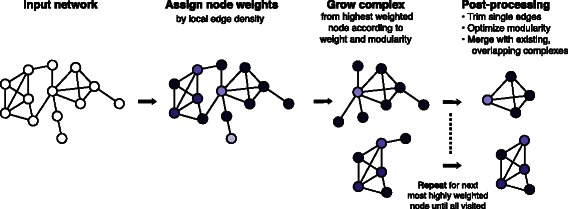
Conceptual Overview of MINE Procedure

### Scoring

MINE receives as input any number of interaction files. The network is treated as an undirected, unweighted graph. Each vertex *v* in the graph *G* = (*V*, *E*) is then weighted based upon its local neighborhood *N*, defined as the set all vertices connected directly to *v* (at a depth of 1); we call the set *N* inclusive of *v* itself {*N*∪*v*}, which we denote simply as *N*∪*v*. The vertex weight (*v*_*w*_) is the product of the maximal number of edges connected to any single node in *N*∪*v* (*k*_*max*_) and the density of *N*∪*v* (*d*): *v*_*w*_ *= k*_max_* d. Density is calculated as *d =* 2 *e*_*N*∪*v*_/(*V*_*N*∪v_* (*V*_*N*∪*v*_*-* 1)), where *V*_*N*∪*v*_ is the number of vertices in *N*∪*v* (i.e. *v* and its direct neighbors) and *e*_*N*∪*v*_ is the number of edges in *N*∪*v*. A cluster (*C*) is then established by iterating through each vertex in *N*∪*v*, in order of highest to lowest weight, and adding neighbors if either of two criteria are satisfied: A) the neighbor vertex weight is above a minimum threshold (as determined by the user-defined vertex weight percentage (*vwp*) of the seed vertex) and does not decrease the cluster modularity score (by an amount equal to or greater than the user-defined modularity score percentage (*msp*)); B) the modularity score for the cluster is improved by *msp*. Cluster modularity (*C*_*mod*_) is defined as the ratio of edges between nodes of a cluster (*E*_*in*_) and edges between cluster members and non-members (*E*_*out*_): *C*_*mod*_ *= E*_*in*_*/E*_*out*_. The process is performed for all vertices in *N*∪*v*, and the resulting cluster can be optionally refined by removing all vertices with *k* = 1 (if the flag *Trim* is set). By default, clusters are non-exclusive (i.e. members are allowed to participate in several clusters); a new cluster will be merged with any existing cluster if their vertices overlap by >50 %, otherwise it will be stored as a new cluster. Each (new or merged) cluster is then scored (*C*_*s*_) as the product of its density (*d*) and the number of members in the cluster (*V*_*C*_): *C*_*s*_ = d * V_*C*_. The cluster identification process is repeated for each vertex in *V* in order of descending *v*_*w*_. The final set of clusters is evaluated for improvements in modularity scores when individual members are excluded, and the membership of each cluster is updated accordingly (by removing nodes from a cluster if the modularity score increases by more than *msp* in its absence).

### Algorithm

1. Vertex Weighting
  procedure Vertex-Weighting
    input: graph: *G* = (*V,E*)
    for all *v* in *G*
      *N* = set of immediate neighbors of *v* (depth = 1)
      *k*_*max*_ = maximum number of edges from any one vertex in set *N*∪*v*
      *d* = density of *N*∪*v*
      *v*_*w*_ = weight = *k*_max_* d
    end for
  end procedure
2. Cluster Prediction
  procedure Cluster-Prediction
    input: graph: *G* = (*V,E*); vertex weight: *v*_*w*_; vertex weight percentage: *vwp*; modularity score
      percentage: *msp*; merge percentage: *mp*
    for *v* ∈ *V*_*w*_ (from high → low weight)
      *N* = set of immediate neighbors of *n* (depth = 1)
      push (*tocheck*, *v* )
      push (*tocheck*, *N* )
      for n ∈ *tocheck*
        if *v*_*w*_ of *n* ≥ (*v*_*w*_ of *v*)(1 – *vwp*) then
          if modularity-score(*C*∪*n*) > modularity-score(*C*) - modularity-score(*C*)*msp then
            add *n* to cluster *C*
        else if modularity-score(*C*∪*n*) > modularity-score(*C*) + modularity-score(*C*)*msp
          add *n* to cluster *C*
        end if
      end for
      if trim == true then call: Trim (*C*)
      if percent overlap C with existing cluster(s) ≥ *mp*
        Merge(*C*) with existing cluster(s)
      *C*_*score*_ = density(*C*) * sizeof(*C*)
    end for
    for C ∈ *AllClusters*
      ModularityCleanup(*C*)
    ends for
  end procedure
  procedure ModularityCleanup
    input: cluster: C
    *v*_*r*_ = *v* that maximizes modularity-score({*C* \*v*})
    while ( sizeof(*C*) > 3 and (modularity-score({*C* \ *v*_*r*_ }) > modularity-score(*C*) + modularity-score(*C*)*msp ) )
      remove *v*_*r*_ from *C*
      *v*_*r*_ = *v* that maximizes modularity-score({*C* \*v*})
    end while
  end procedure
  procedure Trim
    input: cluster: *C*
    for all *v* in *C*
      if *k* of *v* < 2 then remove *v* from *C*
    end for
  end procedure
  procedure modularity-score
    input: cluster: *C*
    *in* = number of edges exclusively between members of *C*
    *out* = number of edges exclusively between members and non-members of *C*
    score = in/out
  end procedure

## Additional file

Additional file 1: Figure S1. Expanded Conceptual Overview of MINE Procedure. The general procedure for the core MINE algorithm is presented with an example input network, vwp = 0.4 and msp = 0.9. The clustering procedure begins with the highest weighted node, where node weights are assigned as *v*_*w*_ = *k*_*max*_ * *d* ( *v*_*w*_ = vertex weight, *k*_*max*_ = largest edge count within node’s local neighborhood, *d* = density ). Nodes are numbered in order visited. Current clusters (blue) are compared to candidate clusters (orange). A node is added to a growing cluster if it passes either of the following criteria: 1) its *v*_*w*_ is within the specified range, or 2) the new cluster modularity (*m*) is within the specified range. The values of *v*_*w*_ and *m* for current and candidate clusters are indicated below each illustrated step; a check mark (or “x”) is placed next to each value if addition of the new node passes (or fails) the corresponding test. After all possible candidate nodes are visited, the preliminary cluster is processed to remove singly-connected nodes and merged with existing clusters if amount of overlap meets the user-defined threshold. (PDF 338 kb)
