# Supplementary material for: MINE: Module Identification in Networks
Source: BMC Bioinformatics. 2011 May 23;12:192. doi: 10.1186/1471-2105-12-192 (PMC3123237; doi:10.1186/1471-2105-12-192)
Supplement: Additional file 1 — Supplementary Figures 1-4 and Supplementary Table 1 in PDF format. [file 1471-2105-12-192-S1.PDF]

Supplementary Figures

Supplementary Figure 1 - Expanded Conceptual Overview of MINE Procedure

The general procedure for the core MINE algorithm is presented with an example input network. The clustering procedure begins with the highest weighted node, where node weights are assigned as $v_w = k_{max} * d$ (v_w = vertex weight, k_{max} = largest edge count within node's local neighborhood, d = density). Nodes are numbered in order visited. Current clusters (blue) are compared to candidate clusters (orange). A node is added to a growing cluster if it passes either of the following criteria: 1) its v_w is within the specified range, or 2) the new cluster modularity (m) is within the specified range. The values of v_w and m for current and candidate clusters are indicated below each illustrated step; a check mark (or "x") is placed next to each value if addition of the new node passes (or fails) the corresponding test. After all possible candidate nodes are visited, the preliminary cluster is processed to remove singly-connected nodes.

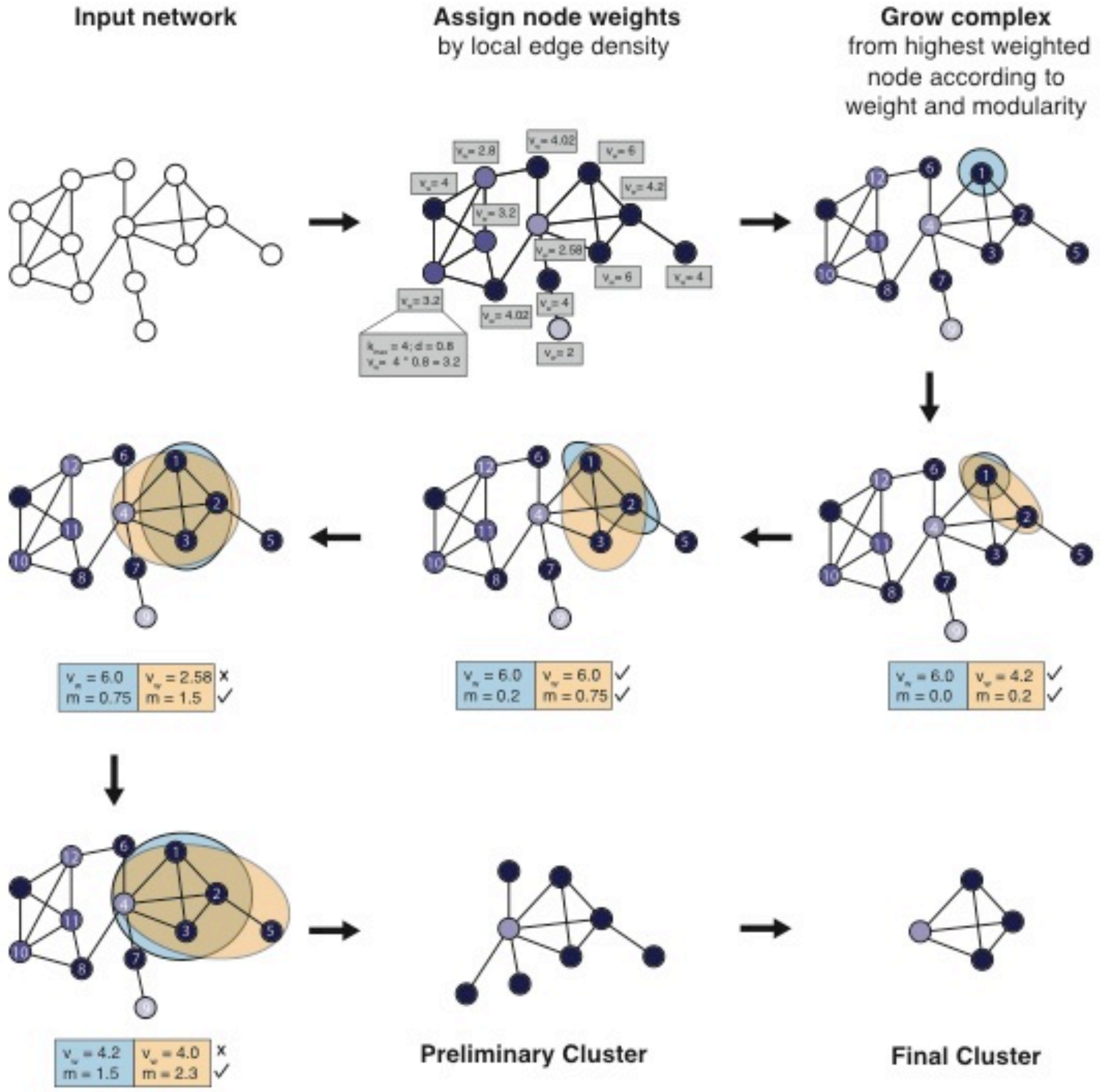
Input network
Assign node weights
by local edge density
Grow complex
from highest weighted
node according to
weight and modularity
Preliminary Cluster
Final Cluster

Supplementary Figure 2 - Recall and Precision of Predicted Clusters with respect to Annotated Complexes

All five algorithms were executed over a broad range of settings, and the recall and precision of clusters with respect to MIPS annotated protein complexes (for yeast) or GO term categories (for yeast and worm) were plotted. Only complexes or categories contained within the networks were considered for calculation of recall and precision. Each data point represents the average recall and precision of all clusters that were a best match with an individual complex, as identified by an algorithm at a specific setting. Thus, for each annotated complex with members represented in the network, the cluster with the most significant overlap (based on a hypergeometric test) was chosen, and the summed scores divided by the total number of annotated complexes. A-B) Yeast FYI network: A) MIPS complexes, B) GO Macromolecular Complexes. C-D) BioGRID yeast two-hybrid network: C) MIPS Complexes, D) GO Macromolecular Complexes. E-G) *C. elegans* interactome from WI8: E) GO Molecular Function categories, F) GO Biological Process categories, G) GO Cellular Component categories. H-J) *C. elegans* interactome from MINT: H) GO Molecular Function categories, I) GO Biological Process categories, J) GO Cellular Component categories. K-M) *C. elegans* interactome from IntAct: K) GO Molecular Function categories, L) GO Biological Process categories, M) GO Cellular Component categories.

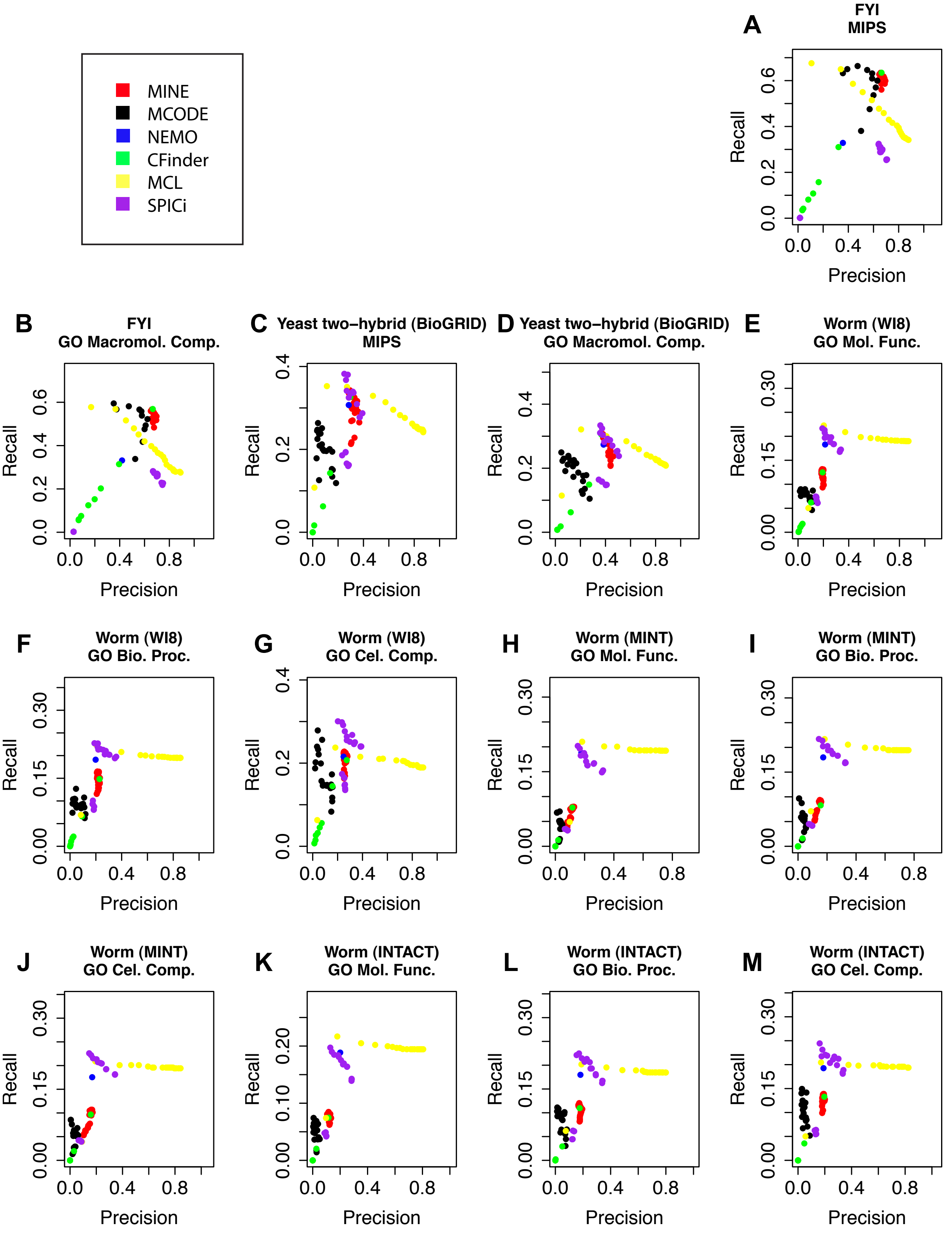
MINE
MCODE
NEMO
CFinder
MCL
SPICi
A FYI MIPS
B FYI GO Macromol. Comp.
C Yeast two-hybrid (BioGRID) MIPS
D Yeast two-hybrid (BioGRID) GO Macromol. Comp.
E Worm (WI8) GO Mol. Func.
F Worm (WI8) GO Bio. Proc.
G Worm (WI8) GO Cel. Comp.
H Worm (MINT) GO Mol. Func.
I Worm (MINT) GO Bio. Proc.
J Worm (MINT) GO Cel. Comp.
K Worm (INTACT) GO Mol. Func.
L Worm (INTACT) GO Bio. Proc.
M Worm (INTACT) GO Cel. Comp.
Recall
Precision

Supplementary Figure 3 - Global Modularity of Identified Clusters

For each algorithm, global modularity scores (y-axis) were calculated over a range of parameters and plotted against the total number of clusters identified (x-axis). Each data point represents the modularity obtained by an algorithm at a specific parameter setting. A) Yeast FYI network. B) BioGRID yeast two-hybrid. C) *C. elegans* interactome from WI8. D) *C. elegans* interactome from MINT. E) *C. elegans* interactome from IntAct.

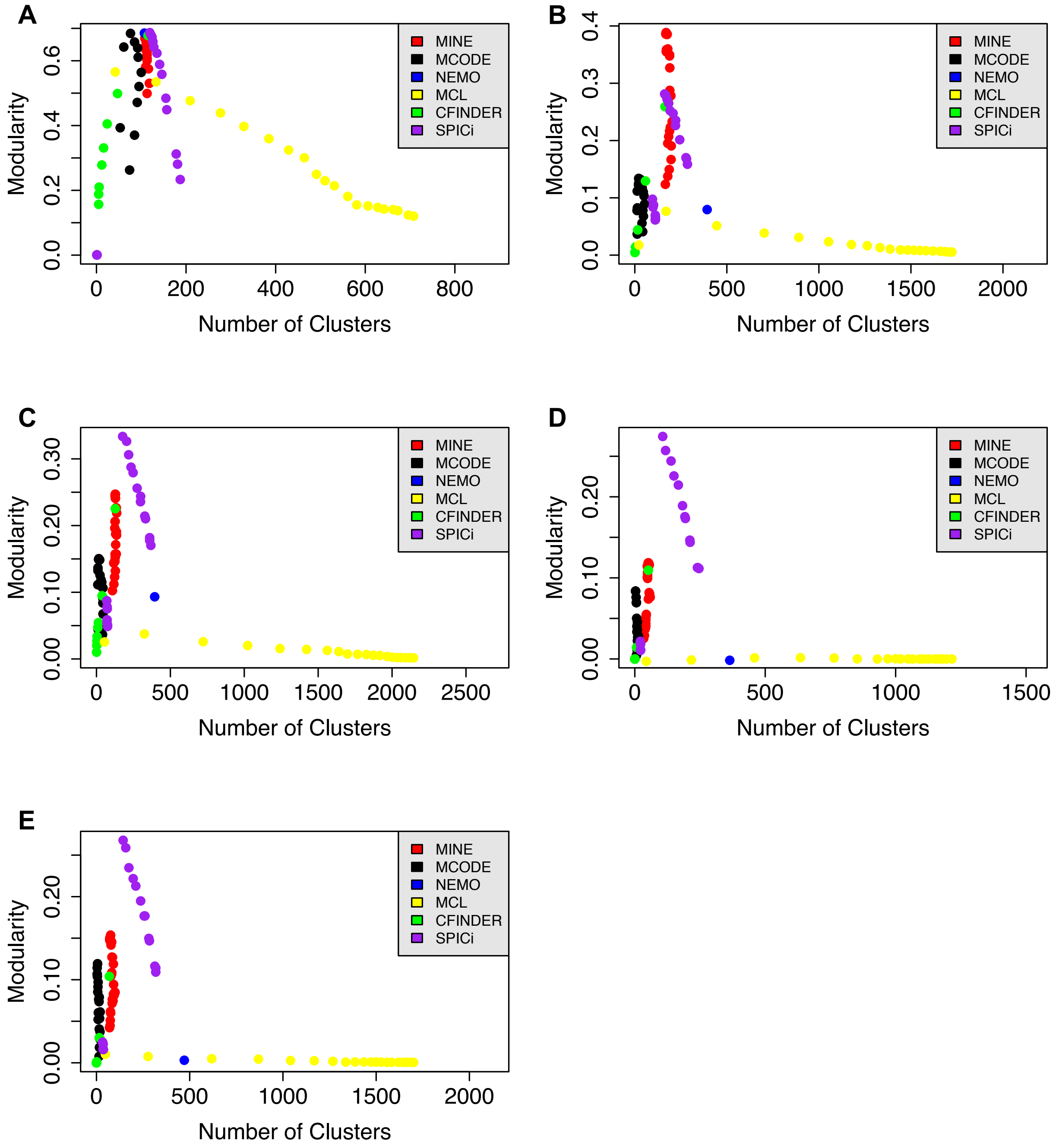

Supplementary Figure 4 - Geometric Accuracy vs. Modularity of Identified Clusters

All five algorithms were executed over a broad range of settings, and the global modularity (y-axis) and geometric accuracy (x-axis) of clusters were plotted. Geometric accuracy was calculated with respect to MIPS annotated protein complexes (for yeast) or GO term categories (for yeast and worm); each data point represents the average value for the most significantly overlapping clusters across all annotated complexes with members in the network. A) *S. cerevisiae* FYI network, evaluated using MIPS complexes. B-C) *S. cerevisiae* yeast two-hybrid network from BioGRID: B) MIPS complexes, C) GO Macromolecular Complexes. D-E) *C. elegans* interactome from WI8: D) GO Molecular Function annotations, E) GO Biological Process annotations. F-H) *C. elegans* interactome from MINT: F) GO Molecular Function annotations, G) GO Biological Process annotations, H) GO Cellular Component annotations. I-K) *C. elegans* interactome from IntAct: I) GO Molecular Function annotations, J) GO Biological Process annotations, K) GO Cellular Component annotations.

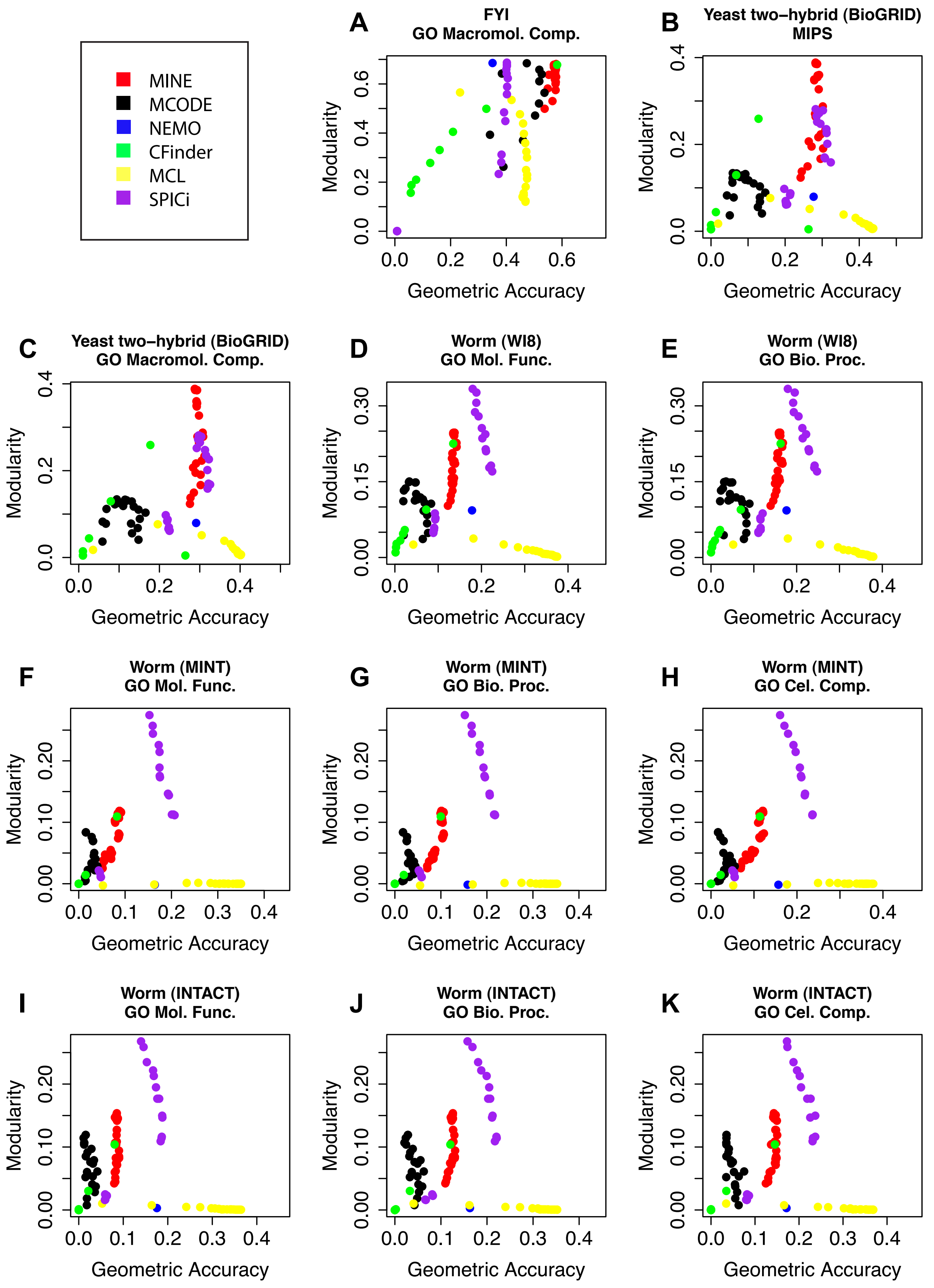

MINE
MCODE
NEMO
CFinder
MCL
SPICi
A
FYI
GO Macromol. Comp.
B
Yeast two−hybrid (BioGRID)
MIPS
C
Yeast two−hybrid (BioGRID)
GO Macromol. Comp.
D
Worm (WI8)
GO Mol. Func.
E
Worm (WI8)
GO Bio. Proc.
F
Worm (MINT)
GO Mol. Func.
G
Worm (MINT)
GO Bio. Proc.
H
Worm (MINT)
GO Cel. Comp.
I
Worm (INTACT)
GO Mol. Func.
J
Worm (INTACT)
GO Bio. Proc.
K
Worm (INTACT)
GO Cel. Comp.
Modularity
Geometric Accuracy

Supplementary Table 1 - Summary of Cluster Characteristics at Optimal Settings

Relevant settings for each algorithm that we consider to yield cluster results with an optimal balance of modularity, geometric accuracy and cluster number. Mean cluster size, density, and geometric accuracy are provided along with standard deviation. Modularity is a composite score over all reported clusters.

Method	Settings	Cluster Number	Mean Cluster Size	Mean Cluster Density	Modularity	Mean Geometric Accucracy
**MINE**	vwp = 0.9; mod = 0.3; trim = *yes*	127	7.89 ± 9.64	0.76 ± 0.28	0.247	0.21 ± 0.17
**MCODE**	vwp = 0.30; haircut = *yes*	44	10.75 ± 18.7	0.76 ± 0.31	0.107	0.14 ± 0.17
**MCL**	gran = 1.4	324	12.04 ± 17.02	0.01 ± 0.02	0.038	0.18 ± 0.07
**Cfinder**	k = 3	127	6.81 ± 19.28	0.86 ± 0.21	0.225	0.21 ± 0.17
**SPICi**	den = 0.25	248	7.09 ± 3.33	0.42 ± 0.23	0.279	0.29 ± 0.15
**NEMO**	NA	394	10.06 ± 8.83	0.07 ± 0.21	0.093	0.21 ± 0.14

Supplementary Table 2 - Summary of Cluster Characteristics at Highest Geometric Accuracy Settings

Relevant settings for each algorithm yielding cluster results that maximize geometric accuracy. Cluster size, density, and geometric accuracy are listed as mean and standard deviation. Modularity is a composite score over all reported clusters.

Method	Settings	Cluster Number	Mean Cluster Size	Mean Cluster Density	Modularity	Mean Geometric Accucracy
**MINE**	vwp = 0.5; mod = 0.5; trim = *yes*	135	5.97 ± 4.64	0.80 ± 0.25	0.190	0.23 ± 0.16
**MCODE**	vwp = 0.30; haircut = *yes*	44	10.75 ± 18.7	0.76 ± 0.31	0.107	0.14 ± 0.17
**MCL**	gran = 5.0	2146	1.81 ± 1.3	0.002 ± 0.04	0.002	0.38 ± 0.11
**Cfinder**	k = 3	127	6.81 ± 19.28	0.86 ± 0.21	0.225	0.21 ± 0.17
**SPICi**	den = 0.65	368	3.4 ± 1.36	0.69 ± 0.09	0.170	0.29 ± 0.15
**NEMO**	NA	394	10.06 ± 8.83	0.07 ± 0.21	0.093	0.21 ± 0.14
