# Supplementary material for: Erratum to: MINE: Module Identification in Networks
Source: BMC Bioinformatics. 2016 Feb 17;17:89. doi: 10.1186/s12859-016-0929-5 (PMC4756399; doi:10.1186/s12859-016-0929-5)
Supplement: Additional file 1: Figure S1. — Expanded Conceptual Overview of MINE Procedure. The general procedure for the core MINE algorithm is presented with an example input network, vwp = 0.4 and msp = 0.9. The clustering procedure begins with the highest weighted node, where node weights are assigned as v w = k max * d ( v w = vertex weight, k max = largest edge count within node’s local neighborhood, d = density ). Nodes are numbered in order visited. Current clusters (blue) are compared to candidate clusters (orange). A node is added to a growing cluster if it passes either of the following criteria: 1) its v w is within the specified range, or 2) the new cluster modularity (m) is within the specified range. The values of v w and m for current and candidate clusters are indicated below each illustrated step; a check mark (or “x”) is placed next to each value if addition of the new node passes (or fails) the corresponding test. After all possible candidate nodes are visited, the preliminary cluster is processed to remove singly-connected nodes and merged with existing clusters if amount of overlap meets the user-defined threshold. (PDF 338 kb) [file 12859_2016_929_MOESM1_ESM.pdf]

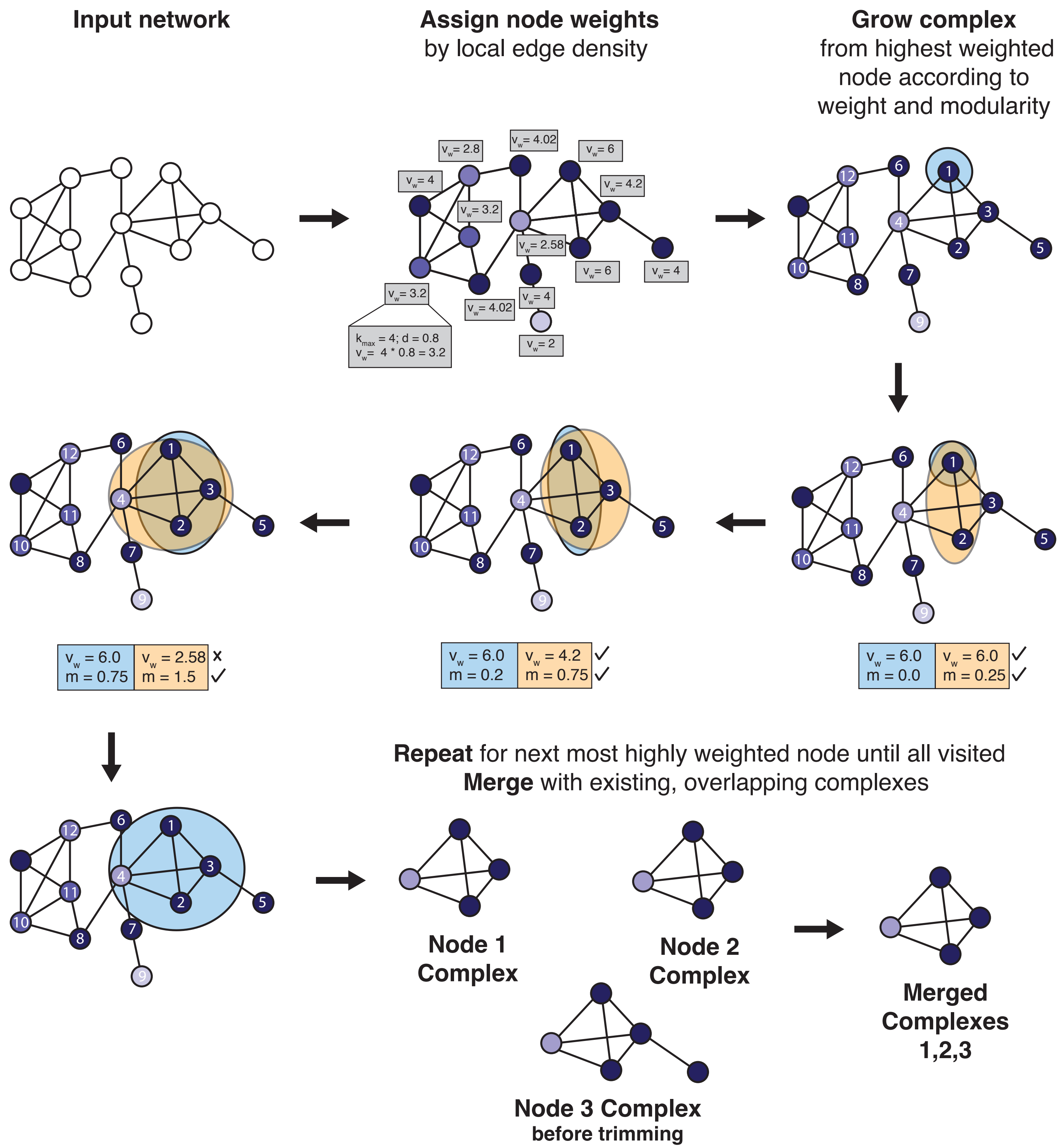

Input network
Assign node weights
by local edge density
Grow complex
from highest weighted
node according to
weight and modularity
v_w = 2.8
v_w = 4.02
v_w = 6
v_w = 4
v_w = 4.2
v_w = 3.2
v_w = 2.58
v_w = 6
v_w = 4
v_w = 3.2
v_w = 4.02
v_w = 4
k_max = 4; d = 0.8
v_w = 4 * 0.8 = 3.2
v_w = 2
v_w = 6.0
m = 0.75
v_w = 2.58
m = 1.5
v_w = 6.0
m = 0.2
v_w = 4.2
m = 0.75
v_w = 6.0
m = 0.0
v_w = 6.0
m = 0.25
Repeat for next most highly weighted node until all visited
Merge with existing, overlapping complexes
Node 1
Complex
Node 2
Complex
Node 3 Complex
before trimming
Merged
Complexes
1,2,3
